# Polycrystalline-Diamond MEMS Biosensors Including Neural Microelectrode-Arrays

**DOI:** 10.3390/bios1030118

**Published:** 2011-08-15

**Authors:** Michael W. Varney, Dean M. Aslam, Abed Janoudi, Ho-Yin Chan, Donna H. Wang

**Affiliations:** 1 Electrical and Computer Engineering Department, Michigan State University, 2120 Engineering, East Lansing, MI 48824, USA; E-Mail: aslam@msu.edu (D.M.A.); 2 Department of Medicine, Michigan State University, East Lansing, MI 48824, USA; E-Mails: janoudi@msu.edu (A.J.); wangdo@msu.edu (D.H.W.); 3 Device Fabrications, MPT, Hong Kong Applied Science and Technology Research Institute Company Limited, 1st Floor, Photonics Centre, 2 Science Park East Avenue, Hong Kong Science Park, Shatin, New Territories, Hong Kong; E-Mail: chanho1@msu.edu (H.-Y.C.)

**Keywords:** biosensors, diamond, electrochemistry, neural probes

## Abstract

Diamond is a material of interest due to its unique combination of properties, including its chemical inertness and biocompatibility. Polycrystalline diamond (poly-C) has been used in experimental biosensors that utilize electrochemical methods and antigen-antibody binding for the detection of biological molecules. Boron-doped poly-C electrodes have been found to be very advantageous for electrochemical applications due to their large potential window, low background current and noise, and low detection limits (as low as 500 fM). The biocompatibility of poly-C is found to be comparable, or superior to, other materials commonly used for implants, such as titanium and 316 stainless steel. We have developed a diamond-based, neural microelectrode-array (MEA), due to the desirability of poly-C as a biosensor. These diamond probes have been used for *in vivo* electrical recording and *in vitro* electrochemical detection. Poly-C electrodes have been used for electrical recording of neural activity*. In vitro* studies indicate that the diamond probe can detect norepinephrine at a 5 nM level. We propose a combination of diamond micro-machining and surface functionalization for manufacturing diamond pathogen-microsensors.

## 1. Introduction

Research on the development of biosensors for neural recording and stimulation, electrochemical detection, and pathogen detection has been ongoing for many years [1,2]. Recently, interest in the use of diamond for biosensors has increased due to its unique combination of properties, including chemical inertness, corrosion resistance, hardness, wide potential windows, feasibility of surface modification and lack of toxic and carcinogenic effects on humans and animals. Polycrystalline diamond (poly-C) has been used in applications including: MEMS packaging structures [3,4,5], RFMEMS resonators [6,7,8,9,10], BioMEMS [11,12], piezoresistive sensors [13,14,15,16], temperature sensors and heaters [17,18], gas/chemical sensors [19,20,21], field emission devices [22,23,24] and microfluidic channels [25,26]. Poly-C and Ultrananocrystalline diamond (UNCD) [27] are viable for use as biocompatible materials in biosensors and other implantable devices used in cardiovascular, neural, muscular, epidermal, orthopedic and other applications.

Diamond has long been used in neural probing by depositing polycrystalline diamond or diamond-like carbon (DLC) on the tips of microwires for implantation in living tissue. Recently, the increased complexity of diamond MEMS allowed the creation of arrays of poly-C electrodes with fixed dimensions and spacing [28]. This technology can be scaled to three-dimensional arrays required for complex applications such as neuroprosthesis. Additional technologies can be combined with diamond microfabrication to create integrated microsystems for biosensing applications beyond neuroscience, such as electrochemical sensing and protein and DNA detection and quantification.

**Figure 1:**
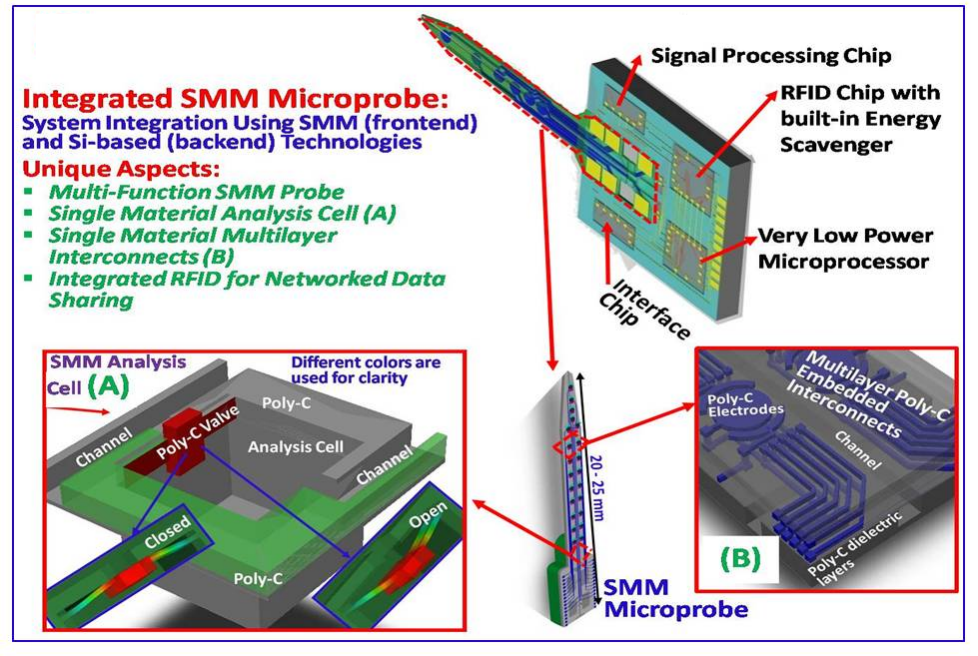
Concept diagrams for multi-functional integrated microsystems for biosensing.

The development of single material MEMS (SMM) technology [29] is expected to lead to new multi-functional probes (Figure 1) that can record individual neuron activity and detect extracellular neurotransmitters. The integrated SMM probes are expected to lead to new generations of healthcare systems using poly-C, UNCD and DLC.

As conventional pathogen detection methods are slow [2], or are not portable [30], methods electrochemical detection of pathogens have been demonstrated [31] that are portable and inexpensive. A UNCD-based immunosensor that uses electrochemical detection of antibody-antigen binding [32] could conceivably be used for pathogen detection using pathogen-specific antibodies. More recently, boron-doped diamond electrodes were used to electrochemically detect the presence of the enzyme beta-galactosidase, which is produced by *E. coli* in response to the chemical isopropyl-B-D-thiogalacto-pyranoside (IPTG) [33]. Such a technique is limited to the detection of specific bacteria and cannot be easily adapted for detection of other pathogenic organisms. Thus, biosensors that are based on immunosensing or DNA detection remain the most flexible and most promising for the development of portable pathogen detection devices.

**Figure 2:**
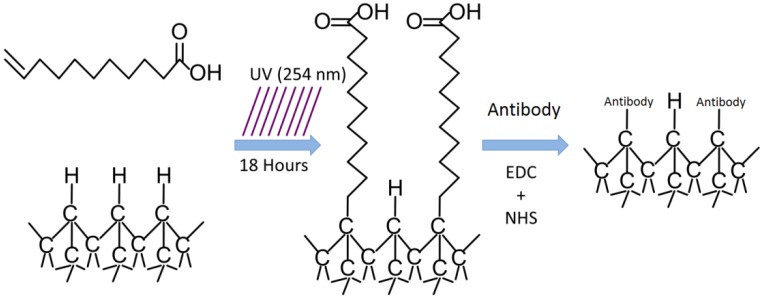
Process flow of diamond functionalization for protein detection.

Diamond-based biosensors that can detect DNA or proteins with a great degree of selectivity and sensitivity require functionalization of the diamond surface with an appropriate molecule (Nucleic acid or protein) that can selectively bind a target molecule (also a nucleic acid or protein); an example can be seen in Figure 2. Other researchers [34,35] have reported poly-C biosensors with a detection limit in the range of 2–10 pM.

One property of diamond that is of particular interest for electrochemical applications is its large potential window (∼4 V). Diamond has been used in many electrochemical detection applications, as can be seen in Table 1 [36]. For biomedical applications, diamond has been used to detect neurotransmitters, such as: dopamine [37,38,39], norepinephrine [37,40], epinephrine [37], serotonin [41,42,43], histamine [43], nicotinamide adenine dinucleotide (NADH) [44] and adenosine [45].

**Table 1:** Substances electrochemically detected using diamond electrodes.

Organic Substances	Inorganic Substances
adenosine, ascorbic acid, caffeine, carbamate pesticides, catecholamines, cephalexin, chlorophenols, chlorpromazine, p-cresol, cysteine, dopamine, formaldehyde, flavonoids, glucose, glutathione, guanosine, histamine, indoles, NADH, nitrophenol, nucleic acids, oxalic acid, penicillamine, phenol, polyamines, purine, pyrimidine, serotonin, sulfa drugs, tetracycline antibiotics, theobromine, theophylline, tiopronin and xanthine	azide anion, hydrazine, hydrogen peroxide, iodide, nitrate, nitrite, dissolved oxygen, dissolved ozone, peroxodisulfate, sulfate, sulfide, Ag^+^, As(III), Cd^2+^, Cu^2+^, Hg^+^, Mn^2+^, Ni^2+^, Pb^2+^, Sn^4+^ and Zn^2+^

Other studies on capillary electrophoresis to detect neurotransmitters using doped-diamond have revealed a detection limit down to 10 nM [37,38]. Sarada *et al*. [43] explored the electrochemistry of histamine and serotonin with boron-doped diamond using cyclic voltammetry, hydrodynamic voltammetry and low inject analysis. The boron-doped poly-C electrode in histamine had a signal-to-noise ratio (SNR) an order-of-magnitude greater than that of the glassy carbon electrode, a linear dynamic range of 3–4 orders of magnitude and a detection limit of about 1 µM. Comparatively, they found the detection limit of 5-hydroxytryptamine was about 10 nM.

Rao *et al*. [44] studied nicotinamide adenine dinucleotide (NADH) using boron-doped poly-C. They found a lower detection limit of 10 nM, while maintaining a SNR of at least seven. Furthermore, they were able to distinguish between NADH and ascorbic acid when the concentrations were comparable. Herlambang *et al*. [41] detected neurotransmitters using an overoxidized poly pyrrole-modified, boron-doped-diamond microfiber electrode. They found detection limits of 500 fM for dopamine and 600 fM for serotonin.

Many studies have investigated the biocompatibility of DLC [46], but few studies have investigated the biocompatibility of diamond. Tang *et al*. [47] found that the biocompatibility of poly-C was similar to that of titanium and 316 stainless steel, which are commonly used in manufacturing implantable devices. Chong *et al*. [48] reported that normal human dermal fibroblast cells were able to attach and grow on the surface of either UV-treated or undecylenic acid-functionalized poly-C, and UNCD was found to be more biocompatible than microcrystalline diamond.

## 2. Development and Fabrication of Poly-C Micro-Electrode Arrays

### 2.1. Development of Poly-C Neural Recording Probes

Neural probes have great potential for use in neuroprosthesis [49,50] and the treatment of neurological disorders [51]. Over the past half-century, neural probes have evolved from individual insulated wires to precision-micromachined, three-dimensional arrays of electrodes (for a review of early neural micro-electrode arrays (MEAs), see Kovacs *et al*. [52]). Modern neural probes are fabricated from a variety of materials, the most popular being silicon and polymers such as polyimide, SU-8 and Parylene C.

Many research groups are developing unique processes for creating neural probes [52,53,54,55,56,57,58,59,60,61,62,63,64]. However, silicon can be an undesirable probe material as, without modification, it has been found to have poor flexibility, solubility in water, and can induce undesirable glial responses [65]. Many groups have solved this problem through coating silicon with biocompatible materials. As an alternative to coating silicon, undoped diamond has been explored as a substrate material due to its structural properties and high biocompatibility [47].

Probes often have different materials serving as electrodes. All neural probes use an inert, metallic (or metalized) substance as their electrodes. Common electrode materials for electrical sensing applications include gold, platinum and iridium. Iridium-oxide electrodes and boron-doped poly-C electrodes have both been shown to be effective for the detection of neurotransmitters [66,67]. Boron-doped poly-C electrodes could potentially be used for both electrochemical detection and for recording electrical signals.

Chan *et al*. [28,68] developed a process for fabricating neural probes using undoped poly-C as the structural material and boron-doped poly-C as the electrode material. Non-diamond materials are used for interconnects and electrical-insulation layers. This probe has been used to measure electrical neural activity, as well as to electrochemically detect neurotransmitters *in vitro*.

### 2.2. Development of All-Diamond Neural Recording Probes Using the Single Material MEMS (SMM) Concept

Ongoing work is focused on the development of a true all-diamond neural probe process using a single material MEMS (SMM) concept [69,70,71]. Through SMM, the fabrication cost can be reduced by using as few masks and materials as possible. Poly-C is good candidate for SMM because of its unique combination of physical properties and the feasibility of selectively growing poly-C to be a semiconductor or insulator.

**Figure 3:**
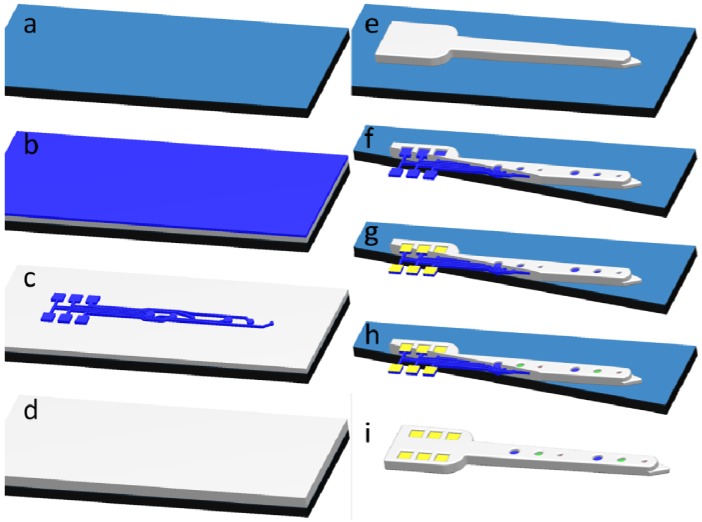
Fabrication process for all-diamond neural probes. (**a**) Si/SiO_2_ substrate; (**b**) Undoped- and doped-diamond growth; (**c**) Doped-diamond etch to define pads, interconnects and electrodes; (**d**) Undoped-diamond growth; (**e**) Undoped-diamond etch to pattern probe shape; (**f**) Undoped-diamond etch to expose pads and electrodes; (**g**) Gold bonding-pad deposition; (**h**) Optional diamond functionalization; (**i**) Probe release in HF.

**Figure 4:**
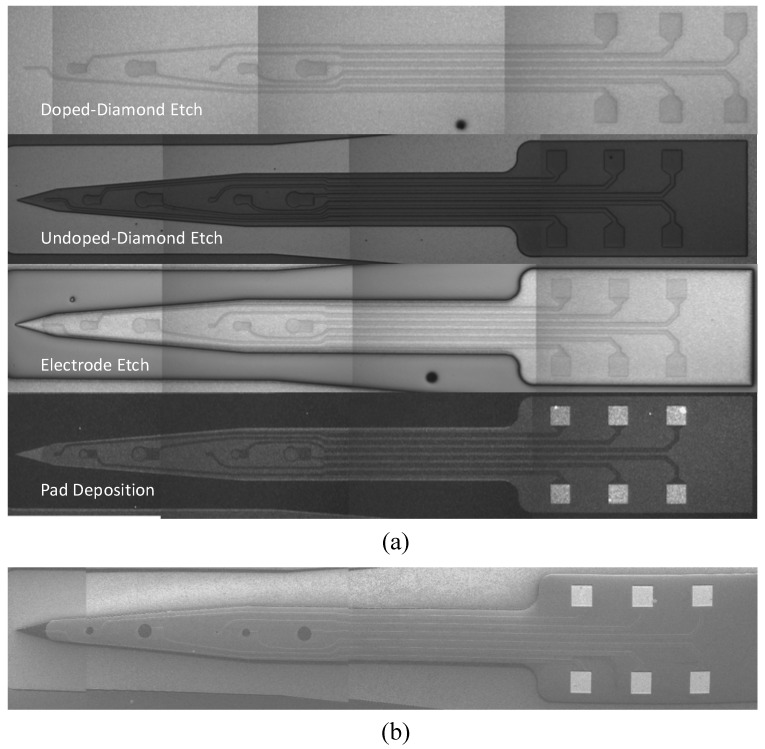
Micrographs of all-diamond neural probe fabrication. (**a**) Optical micrographs showing key fabrication steps; (**b**) Scanning electron micrograph showing the finished probe before release in HF.

The SMM concept can be applied to neural probe fabrication. Neural probes can be fabricated using as few as three or four masks and one material. In the all-diamond probe, undoped diamond (10^7^ Ω-cm) serves not only as a structural layer, but also as the insulating layer. Highly boron-doped diamond (10^3^ Ω-cm) is utilized for interconnects and electrodes. The layers are patterned using CF_4_ reactive ion etching (RIE). The fabrication process for all-diamond probes is illustrated in Figure 3, and micrographs of probes being fabricated can be seen in Figure 4.

The first step in the poly-C MEA fabrication process is diamond growth. All of the diamond growth is done using MPCVD at a power of 2.3 kilowatts. Diamond is grown in hydrogen plasma with methane used as the carbon source. When an undoped diamond film is desired, oxygen is added to the growth environment. The presence of oxygen in the plasma correlates to an increase in the resistivity of the diamond. To achieve conductive diamond, trimethylboron (B[CH_3_]_3_) diluted in H_2_ is added to the growth environment. Before diamond growth can occur, the substrate must be seeded with nano-sized diamond particles. DI water loaded with diamond powder is spun at 4,000 RPM onto a silicon wafer with ten microns of silicon dioxide. The first layer of diamond grown is an undoped layer. Growing diamond from seeds with oxygen in the growth environment can result in undesirable crystal morphologies. To avoid this, a layer of diamond is grown with only hydrogen and methane. After a continuous diamond film has been grown, oxygen is added to the plasma so that the ratio of hydrogen to methane to oxygen in the growth environment is roughly 200:1:0.25. After the diamond deposition reaches about four microns, oxygen flow is stopped and trimethylboron is added to the growth environment to grow a layer of boron-doped diamond. The ratio of hydrogen to methane to trimethylboron during doped diamond growth is approximately 200:1:0.2. After a one micron thick doped-diamond film is grown, the first diamond growth step is complete.

Next the doped-diamond layer is selectively etched to form what will become the electrodes, interconnects and backend bonding pads. Etching is done using RIE with CF_4_ plasma with aluminum as a masking layer. During the etching process, the peaks of the original faceted structure are smoothed without diamond columns (needles or whiskers) developing. The overall surface roughness is reduced by 50% at most, as measured by profilometer. [69] indicates that the highest etch rate of poly-C (~300 Å/min) and the highest selectivity of poly-C to Al (11:1) in CF_4_ plasma are both achieved at a chamber pressure of 80 millitorr. By adding argon to the CF4 plasma, the etch rate of poly-C can be improved by 20% at a RF power of 300 watts.

A top layer of undoped diamond is grown to act as the insulating layer. The growth recipe for this layer is the same as before except there is no need to begin growth without oxygen as diamond is being grown from a preexisting diamond layer. The thickness of the deposited diamond film is about one micron, making the total probe thickness about 6 µm.

The shape of the probes is defined in the next step. CF_4_ RIE is used again to etch through both undoped diamond layers, the silicon dioxide layer, and into the silicon layer. Next the electrodes and backside pads are exposed through RIE of the top undoped diamond layer. The electrode sizes varied from 5 through 150 µm in diameter. Then chromium and gold are deposited onto the backend pads as a precursor step for probe bonding.

At this point, the electrodes can be selectively modified to achieve different electrode characteristics. Diamond crystals can be terminated by a variety of elements or compounds. Some of the most common, and most stable, terminations of diamond include hydrogen, oxygen and fluorine. Hydrogen, oxygen and fluorine terminations were achieved by brief exposure to H_2_, O_2_ and CF_4_ plasma, respectively. Each of these terminations will result in different electrical properties of the electrode because of the difference in interactions between the electrode surface and solution. This can potentially be used to create different types of electrodes from the same material. This proves to be very useful when using probes for electrochemical measurements.

The final step in probe fabrication is release. The sacrificial silicon dioxide layer is wet etched in hydrofluoric acid. The diamond layers are unaffected by the HF etch and the etch rates of Au and Cr are negligible. Poly-C MEAs have been fabricated using the SMM concept with only four masks used for electrical recording probes and six masks used for electrochemical probes.

For testing the fabricated probes, they were bonded to a printed circuit board. This allowed easy connections for both *in vivo* and *in vitro* experiments. In the future, post processing of the probes could also enable them to be incorporated into a wireless microsystem for neural interfaces. SMM diamond packaging which could be done during the probe processing has also been explored [70].

## 3. Experiments and Results

### 3.1. *In Vivo* Electrical Neural Recordings

**Figure 5:**
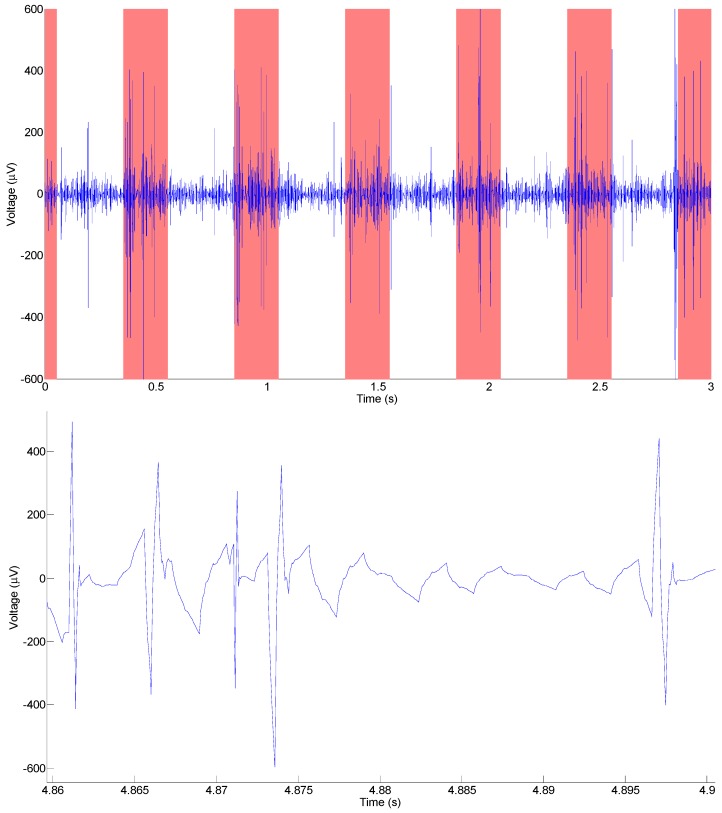
Neural recording from the audio cortex of a guinea pig taken with a neural probe with a diamond electrode. In the top graph, the red bars indicate where auditory stimulus was applied. The bottom graph shows a close up of several action potentials.

The Michigan State University (MSU) neural probe, based on poly-C, was successfully tested as an extracellular, *in vivo* neural recording device [12]. In 2008 a MSU neural probe was surgically implanted into a live guinea pig’s auditory cortex, which marked the first implantation of a diamond-based neural probe into a living animal. In the experiment, the anesthetized guinea pig was subjected to 200 ms, wide-frequency audio-pulses at a frequency of 2 Hz. Figure 5 shows an example of a recorded electrical signal after filtering and signal processing.

The recordings show peak values every 500 ms, corresponding to the frequency of the applied stimulus. However, the recorded signals appear to have a lower SNR than what is often presented for silicon-based neural probes with other electrode materials. Low SNR could be due to a myriad of reasons, such as: a large distance between the neurons and electrodes, inappropriately sized electrodes (diameter was 30 µm), high impedance per area of the electrode (electrode impedance of ~70 kΩat 1 kHz) or poor insulation between recording sites (>100 kΩ). The SNR issue can be alleviated through signal processing. The signal in Figure 4 has been improved with a 1-D wavelet denoising. The denoising was performed with hard thresholding using the rigorous SURE method for non-white noise. The discrete wavelet transform was performed, using Debauchier’s second wavelet, at a level of 5.

**Figure 6:**
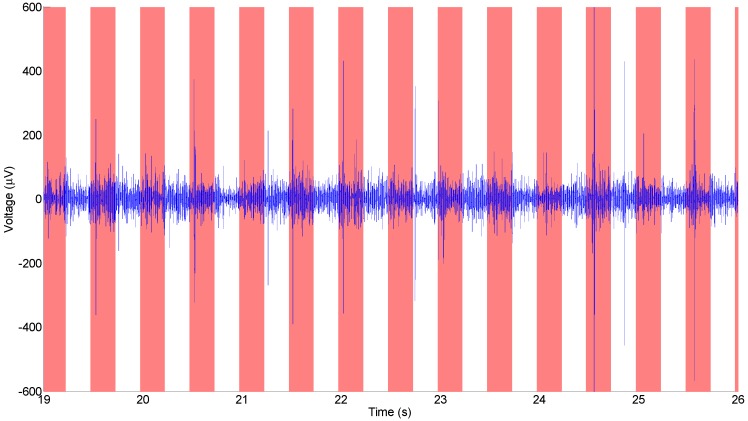
Neural recording from the audio cortex of a guinea pig taken with an all-diamond SMM neural probe, the red bars indicate where auditory stimulus was applied.

**Figure 7:**
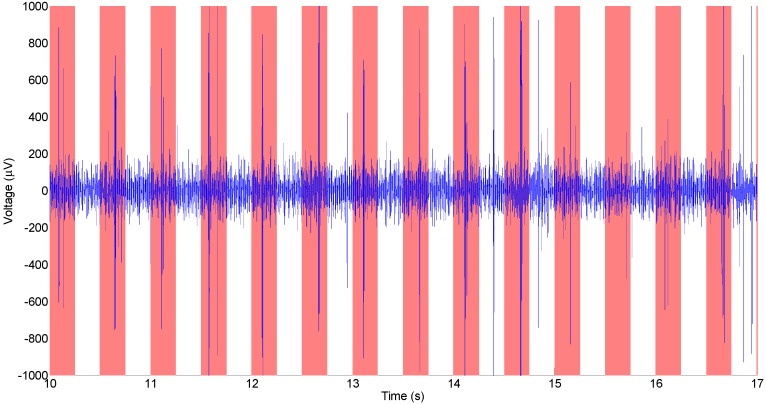
Neural recording from the audio cortex of a guinea pig taken with a silicon-based neural probe, the red bars indicate where auditory stimulus was applied.

This experiment was repeated using SMM neural probes. Figure 6 shows a typical example of a neural recording from an all-diamond electrical recording probe, after signal processing. Results from one electrode are shown, but other electrodes had similar results. The recording shows peak values every half second corresponding to the time intervals between applying stimuli. The SMM neural probes have an SNR which is comparable to other diamond electrodes (e.g., Figure 5), but slightly worse. The slightly poorer SNR can be accounted for by the lower conductivity of doped diamond, as compared to metals typically used for probe interconnects. For comparison, a recording performed using a silicon-based probe with platinum electrodes can be seen in Figure 7.

### 3.2. *In Vitro* Electrochemical Detection with Poly-C Micro-Electrode Arrays

The Michigan State University (MSU) poly-C probes have also been developed for electrochemical detection [12,68,71]. The main difference between these probes and those reported for electrical recording is that there are additional electrode materials used for the counter and reference electrodes. In the initial electrochemical probes, Pt was used as a counter electrode, Ag/AgCl was used for the reference electrode and boron-doped diamond was used for the working electrode. *In vitro* electrochemical detection experiments have been performed using these probes [12]. This first generation of electrochemical neural probes was never tested *in vivo* due to complications arising from the Ag/AgCl reference electrode. The electrode eventually degrades in solution as the Ag is dissolved. This results in a non-functional reference electrode for long-term applications. Furthermore, Ag has known cytotoxic effects, making it not biocompatible. While the first generation of the poly-C electrochemical probe may not be useful for chronic *in vivo* experiments, it has proven useful in demonstrating the efficacy of the probe’s poly-C working-electrode.

**Figure 8:**
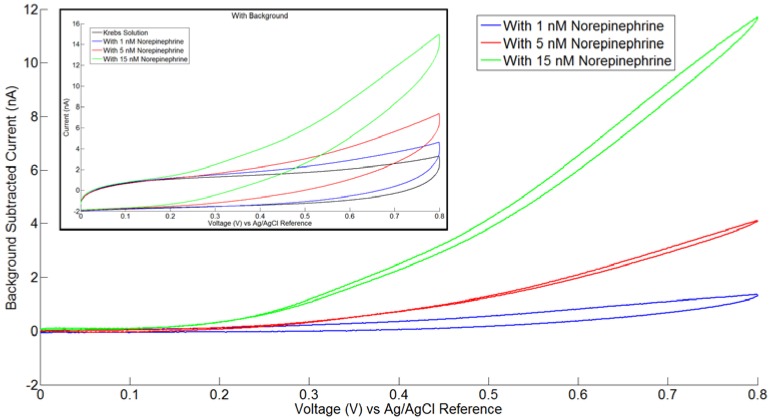
Family of background-subtracted cyclic voltammograms of krebs solution with varying amounts of norepinephrine (NE), demonstrating a lower detection limit of 5 nM or less. The inset shows the cyclic voltammograms before subtracting the krebs solution background.

Initial experiments in neurotransmitter detection indicate a detection limit of approximately 5 nM. Figure 8 shows a family of cyclic voltammograms recorded using the diamond electrochemical probe. The diamond electrochemical probe was used with a commercial Ag/AgCl reference electrode, the working electrode was boron-doped diamond, and the counter electrode was the on-probe Pt electrode. In the experiment, norepinephrine (NE) was added to krebs solution. The figure shows that there is a clear difference between 1 nM, 5 nM and 15 nM, indicating a quantification limit in the 5 nM range. This detection limit is consistent with other results using diamond as a working electrode [37,38,43,44]. The figure also demonstrates an extremely low background current, a major advantage of the diamond electrode. Performing this cyclic voltammetry with other fabricated diamond probes has comparable results. The cyclic voltammetry method was chosen to test the electrochemical probe because it is easy to interpret and is applicable to testing for small concentrations of analytes.

**Figure 9:**
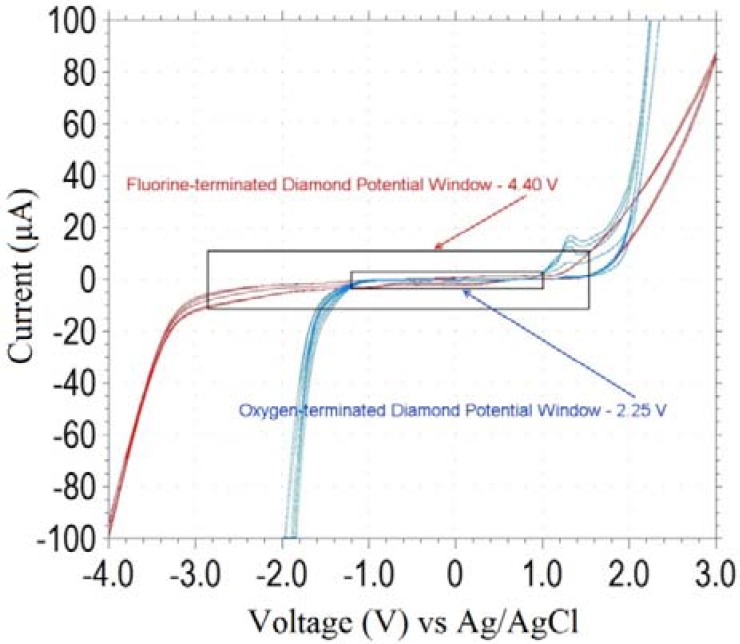
Cyclic voltammogram comparing the potential windows of oxygen-terminated diamond and fluorine-terminated diamond in 1 M KCl.

It has also been found that the potential window of the poly-C working-electrode can be altered significantly by modifying the surface of the poly-C. Fluorine-terminated diamond has a much greater potential window than oxygen terminated diamond. As seen in Figure 9, the potential window of a fluorine-terminated electrode extends much further on the reduction side of the voltammogram. This means that more chemicals could be detected by means of reduction using this electrode. Furthermore, the true potential window of the electrode cannot be measured using a KCl solution. The oxidation reaction is limited by the oxidation of Cl^−^ in solution, known to be at 1.36 V, and the reduction reaction is limited by the reduction of K^+^ in solution, known to be at −2.93 V. These reactions result in the potential window of KCl to be 4.29 V, which is achieved by the electrode.

## 4. Conclusion

Diamond's unique combination of physical and chemical properties and the feasibility of functionalizing its surfaces with nucleic acids and polypeptides make diamond a promising material for use in biosensors that can be used to measure neural activity or to detect and quantify biological molecules and pathogens. Detection limits for diamond biosensors are typically in the fM–nM range. Results on the fabrication and application of diamond neural probes for electrical and chemical detection demonstrate the feasibility of using diamond micro-machining technologies to produce a new generation of pathogen microsensors and microsystems made from poly-C.
